# Exploring barriers and facilitators to integrated policy formulation and implementation of family planning and urban development programmes in Nigeria

**DOI:** 10.1186/s12961-022-00924-0

**Published:** 2022-10-28

**Authors:** Sunday A. Adedini, Blessing U. Mberu, Christiana A. Alex-Ojei, Lorretta F. C. Ntoimo

**Affiliations:** 1 Demography and Social Statistics Department, Federal University Oye-Ekiti, Oye-Ekiti, Nigeria; 2 Demography and Population Studies Programme, Schools of Public Health and Social Sciences, University of the Witwatersrand, Johannesburg, South Africa; 3 African Population and Health Research Center, APHRC Campus, Nairobi, Kenya

**Keywords:** Urban development, Family planning, Reproductive health, Multisectoral actions, Nigeria

## Abstract

**Background:**

As more people now live in urban areas than in rural communities in Nigeria, urban development (UD) requires urgent policy and programmatic attention. Although the population factor has been identified as important to achieving national development goals, and evidence suggests that meeting the family planning (FP) and reproductive health (RH) needs of the vulnerable urban population can serve as an important recipe for achieving population growth rates consistent with building sustainable, habitable and prosperous urban settings, FP remains a neglected subject in UD initiatives in Nigeria. This study explored barriers and facilitators in achieving integrated policy formulation and implementation of FP and UD programmes in Nigeria.

**Methods:**

We conducted key informant interviews (*n* = 37) with relevant FP/RH and UD stakeholders in Ibadan and Kaduna—two megacities that have undergone several UD and FP intervention programmes in the south and north of Nigeria. The sample size was determined by data saturation. Data were organized using ATLAS.ti and NVivo 12 software, and analysis was conducted using a thematic approach.

**Results:**

We found that relevant government agencies largely work in silos. Other identified barriers to integrated policy formulation/implementation of FP and UD programmes in Nigeria include lack of knowledge about the FP–UD nexus between professionals, ineffective implementation and monitoring of existing guidelines, lack of policy documentation that clearly links FP and UD, and frequent transfer of government stakeholders. Notwithstanding the identified barriers, the study established ways of achieving synergy between FP and UD sectors, including stakeholder engagement, intersectoral collaborations, sensitization and publicity, roundtable discussion, interdisciplinary research, conferences and other interactive and knowledge-sharing fora.

**Conclusions:**

We conclude that addressing barriers to the intersectoral linkage between FP and UD is fundamental to achieving sustainable urbanization in Nigeria.

## Background

The world is becoming increasingly urbanized, with more than 54% of the global population currently living in cities [1]. Although cities are hubs for socioeconomic growth and development, many metropolitan areas in low- and middle-income countries lack the appropriate policies and programmes needed to make cities inclusive, safe, resilient and sustainable in line with the United Nations Sustainable Development Goal (SDG) 11 [3]. Africa is projected to record the fastest urban growth in the world. Mills and colleagues [4] noted that urgent action is needed to address the African urban population explosion. Nigeria—which is by far the most populous country in Africa—has an estimated population of 206 million [5, 6]. This figure is projected to increase to 410 million by 2050, and more than 70% of the population will be living in urban areas. Nigeria’s statistical agency has put the country’s annual urban growth rate at 6% [7]. It is a major concern that infrastructure development and service delivery do not keep pace with the increasing urbanization in Nigeria. Municipal authorities often find it difficult to meet the increasing urban population demand for basic services. As a result, a huge proportion of Nigerian urban residents live in slums or slum-like areas with limited access to basic services such as quality education, healthcare, adequate security, safe water, good sanitation and waste management. Consequently, the commonly assumed urban advantage has been challenged in many parts of the developing world, thus raising increasing global concerns as to the viability of urban living and the intricate policy and programme challenges in addressing the scarce livelihood opportunities for the poor and exposure to pernicious health conditions [8]. In the case of Nigeria, the right policies and investments are therefore needed to address the challenges of urbanization and manage Nigeria’s teeming urban population for sustainable development.

In the search for pathways to sustainable urbanization in the sub-Saharan African (SSA) region, the population factor has been identified as important to achieving national development goals, and a corpus of evidence suggests that meeting the family planning (FP) and reproductive health (RH) needs of the vulnerable urban population can serve as an important recipe for achieving population growth rates consistent with building sustainable, habitable and prosperous urban settings [9–12]. Against the backdrop of mounting economic, political and social challenges and the search for key policy pathways to address the determinants of rapid population growth to achieve population thresholds congruent with economic growth and development aspirations in SSA, Mberu and Ezeh [13] demonstrated the implications of different rates of population growth in the push to eradicate different dimensions of extreme poverty and hunger in Zambia and Botswana, highlighting the substantial consensus that FP interventions are consistent with achieving development goals relating to reducing poverty and maternal and child mortality, as well as other salient objectives, such as environmental stability and access to basic services.

Further, a recent study by Harpham and colleagues [14] attempted to link FP and urban development (UD) and thus establish the importance of incorporating the population factor into UD initiatives. Evidence also shows that reducing the fertility rate through effective contraception can help foster socioeconomic growth and healthy urban situations [15, 16]. Nevertheless, FP remains a neglected subject in UD initiatives in Nigeria. As a result, the modern contraceptive prevalence rate (mCPR) remains very low among the urban population in Nigeria—currently estimated at 18.2%, compared with over 70% in countries like Malawi and Rwanda [15].

On the synergy between health and UD, WHO has been increasingly highlighting urban health across sectors, with a focus on how to integrate health into UD and governance to achieve sustainable UD, and how health prominently features in the interlinkages between and among the SDGs, including Goal 11, and the New Urban Agenda on sustainable cities and communities, cutting across almost all others and across traditional policy and disciplinary silos [17]. Consequently, effective implementation of UD strategies would require a multisectoral approach. Unfortunately, the problem of silos in research, policy and programmatic implementation has created a huge gap, thus constituting a barrier to the effective incorporation of FP programmes into UD initiatives. Given that high-fertility families have socioeconomic and health challenges and grave implications for urban poverty, and because an effective FP programme can contribute to the achievement of UD goals in line with the United Nations SDG 11, this study (1) explored barriers to integrated policy formulation and implementation of FP and UD programmes in Nigeria, and (2) investigated how a multisectoral approach and collaborations between FP experts and urban planners could be achieved in Nigeria.

## Methods

### Study design, setting and population

This paper is drafted from a larger study that employed a qualitative methodology. The study was designed as an exploratory enquiry to understand the perspectives of relevant stakeholders on how synergy could be achieved in policy formulation and implementation of programmes on FP/RH and UD in Nigeria.

The target population/participants for the study were key informants purposively selected from among stakeholders working in FP and UD sectors at the state and local government levels in two urban locations in Nigeria—Ibadan and Kaduna. The two cities were purposively selected as they have witnessed many FP intervention programmes such as the Nigerian Urban Reproductive Health Initiative (NURHI), and those implemented by the United Nations Population Fund (UNFPA), Society for Family Health and Marie Stopes International (now MSI Reproductive Choices), among others.

### Participants and recruitment

The research consisted of 37 purposively selected stakeholders in the two cities. See Table 1 for a description of the participants by selected background information. The criteria for selection were that participants were in a key leadership position and possessed professional training and experience in nongovernmental organizations (NGO) working on FP/RH and UD and physical development, or were in relevant government ministries, departments and agencies.

**Table 1:** Profile of the key informants

S/N	Age (years)	Education	Residence	Work type	Designation
FP experts					
1	45	Tertiary	Kaduna	NGO	Regional coordinator
2	68	Tertiary	Kaduna	CSO	Communication officer
3	36	Tertiary	Kaduna	Government hospital	Assistant chief nursing officer
4	53	Tertiary	Kaduna	Government hospital	Chief consultant gynaecologist
5	54	Tertiary	Kaduna	CSO	Health facilities supervisor
6	47	Tertiary	Kaduna	CSO	Executive director
7	63	Tertiary	Kaduna	Government ministry	Commissioner
8	52	Tertiary	Kaduna	Government ministry	RH/FP coordinator
9	38	Tertiary	Kaduna	Government hospital	Medical director
10	52	Tertiary	Ibadan	Government agency	FP coordinator
11	62	Tertiary	Ibadan	NGO	Team lead
12	27	Tertiary	Ibadan	NGO	Programme officer
13	53	Tertiary	Ibadan	Government agency	Executive secretary
14	56	Tertiary	Ibadan	Government hospital	Unit head, programme coordinator
15	59	Tertiary	Ibadan	Government	Chief nursing officer
16	30	Tertiary	Ibadan	NGO	Technical officer
17	54	Tertiary	Ibadan	Government	FP provider
18	56	Tertiary	Ibadan	Government	Chief medical officer of health and director of primary health
Urban planners					
19	62	Tertiary	Kaduna	Government tertiary institution	Consultant/chief lecturer
20	57	Tertiary	Kaduna	Government tertiary institution	Consultant/principal lecturer
21	61	Tertiary	Kaduna	Private practice	Consultant
22	51	Tertiary	Kaduna	Private practice	Consultant
23	60	Tertiary	Kaduna	Government tertiary institution	Consultant/chief lecturer
24	44	Tertiary	Kaduna	Government/consulting	Head, GIS/principal partner
25	49	Tertiary	Kaduna	Government ministry	Chief town planning Officer
26	42	Tertiary	Kaduna	Media organization	Director
27	35	Tertiary	Kaduna	NGO	Head, social entrepreneur
28	51	Tertiary	Ibadan	Government ministry	Acting director
29	43	Tertiary	Ibadan	Private university/NGO	Consultant/associate professor
30	57	Tertiary	Ibadan	Private urban development firm	Director
31	50	Tertiary	Ibadan	Government	Principal town planning officer
32	46	Tertiary	Ibadan	Private practice	Principal partner
33	57	Tertiary	Ibadan	Private town planning firm	Principal partner
34	47	Tertiary	Ibadan	Government tertiary institution	Consultant/lecturer I
35	66	Tertiary	Ibadan	Government tertiary institution	Consultant/associate professor
36	53	Tertiary	Ibadan	Private practice	Consultant
37	46	Tertiary	Ibadan	Private practice	Principal partner

*S/N* serial number, *CSO* civil society organization, *FP* family planning, *GIS* geographic information system, *NGO* nongovernmental organization, *RH* reproductive health

### Data collection and procedures

Data were collected using key informant interviews with selected participants in relevant civil society organizations (CSOs) and NGOs, including NURHI and the International Planned Parenthood Federation (IPPF). Key informant interviews were also conducted with government officials in the Ministry of Health, Ministry of Women Affairs and Youth Development, Ministry of UD and Physical Development, and Ministry of Education, as well as FP units.

A 3-day training was organized to inform the research assistants who served as note-takers about the study objectives and the art of taking notes in a qualitative study. Separate key informant guides for FP experts and urban planners were prepared and pretested. Before the actual data collection, a pilot study was conducted in Oyo Township, a border town to Ibadan. The pilot exercise was followed by a debriefing session which assisted in revising the study tools and processes. The actual fieldwork took place between October and December 2020. The lead investigator conducted the interviews, with additional support such as booking appointments with respondents and note-taking provided by well-trained research assistants who had rich experience in qualitative data collection. First, information was obtained on the socioeconomic and demographic characteristics of study participants. Second, we used semi-structured open-ended qualitative study guides for the interviews. We obtained relevant information on the study objectives, including challenges of the urban settings, and ways of achieving interlinkage between FP and UD.

A total of 37 interviews were conducted, 18 with FP experts (9 in each city) and 19 with urban planners (9 in Kaduna and 10 in Ibadan). The sample size was determined by data saturation. The average duration of the interviews was 55 minutes, and each interview ended when no further issues arose. All interviews were conducted in English, audio-recorded and transcribed verbatim by experienced transcribers. Field notes were taken to support the transcripts.

### Data management and analysis

Data were analysed by experts in qualitative data analysis using computer-aided qualitative data analysis software. All the transcripts were coded using ATLAS.ti 6.2.25 and NVivo 12. The lead investigator validated the data.

The data analysis followed the deductive and inductive approaches to thematic coding. Codes were generated from the interview guides and the project objectives, and the themes emerging from the narratives. Each transcript was coded after reading the transcripts several times to become familiar with the data. Similar codes were merged, and all codes were grouped into subcategories and main themes. The results are presented thematically in narratives with apt quotations using two broad themes drawn from the study objectives. One is the barriers to integrated policy formulation and implementation of FP and UD programmes. This was presented using five subthemes: lack of extant policy, programme or document linking FP and UD; ineffective implementation and monitoring of existing guidelines; poor involvement of policy-makers; frequent transfer of government stakeholders; and perception of no nexus between FP and UD. The second broad theme is a multisectoral approach and collaborations between FP experts and urban planners. This is presented using two subthemes: intersectoral collaboration, and suggestions on ways to achieve multisectoral synergy between FP experts and urban planners. The suggestions were further presented using five subthemes: stakeholder engagement, involving nonprofessional stakeholders, conferences and roundtable discussions, multidisciplinary research and publicity, and sensitization talk with government officials and political leaders.

## Results

Responses from the various stakeholders in FP and UD show that their opinions on the synergy between the two sectors were based on their fields and experience.

### Barriers to integrated policy formulation and implementation of FP and UD programmes

The respondents identified barriers to the integration of FP and UD programmes. They indicated that there is no identifiable policy document or programmes that clearly link FP to UD and physical development. They also identified barriers such as ineffective implementation and monitoring of existing guidelines in each sector, achieving the buy-in of policy-makers, frequent transfer of government stakeholders, inadequate knowledge about policies and programmes that stakeholders consider to be out of their scope of discipline or operation, and perceptions of no nexus between FP and UD.

#### Lack of extant policy documents or programmes linking FP and UD sectors

When asked about the existence of a policy framework or programme that links FP and UD sectors, most responses included “no”, “not aware” or “don’t know”, thus indicating that many of the informants were unaware of any such policy document or programme. However, a few FP experts reported having seen a document developed by NURHI that attempted to establish synergy between FP and UD. One barrier to developing a policy document linking FP to UD as identified by the respondents was the risk of being misconstrued by the people, and being branded as anti-people because of the conservative notions about FP in some parts of Nigeria. Some respondent views were as follows:

*Even if there is a link between the two sectors, I don’t think we have it here. I think it will be in the Ministry of Health because (yes) they have so many documents there.* (FP expert, commissioner, government ministry)

*There is one document I know; I saw it in NURHI office at a time. It speaks about family planning and urban development. It was a case of Kaduna. It was developed between 2015 and 2016. It’s a tool used for advocacy. It’s a model that speaks about what needs to be done that will be part of the investment of the state. For instance, we are looking at if the state would invest 8 billion naira*^1^* on FP, the kind of results it’s going to get looking at population, looking at infrastructure, looking at food security and other things like that.* (FP, executive director, CSO)

*At this stage, I don’t think the Kaduna state government will develop and make that kind of document for the public because of the sensitivity attached to family planning. Once you bring out something like that without being 100% sure of the thinking of the people, the government will be branded antireligious or anti-God because we still have people who are not yet in support of family planning, the term adopted by the Kaduna state government is child-spacing service and not family planning, because it is so sensitive here.* (FP expert, executive director, CSO)

Urban planners also reported that there are no policies that explicitly link FP and UD. Their responses also show limited or lack of knowledge about the FP programme:

*There is no policy or programme linking family planning and physical planning. I don’t think so except we have individuals, and nongovernmental organizations. There is really no synergy between our ministry of physical planning or physical development and family planning for now.* (Urban planner, acting director, government ministry)

*I know that I am aware of the family planning programme at the national level, but linking it with UD … I can’t say, I don’t know anything like that.* (Urban planner, consultant, government institution)

#### Ineffective implementation and monitoring of existing guidelines

Another barrier identified by the respondents is a weak implementation and monitoring mechanism. For instance, some urban planners reported that there were physical planning laws (such as the housing standards law) that specified the appropriate number of individuals who should be in a standard room. Urban population explosion would have been avoided in many Nigerian cities if these laws had been well enforced at the national and subnational levels. However, these standards and regulations were not enforced, as there is a recognized lack of monitoring mechanisms by the statutory government agencies. Two urban planners expressed the following views:

*There is this standard that has been stipulated for long, the number of people that should occupy a home, especially a normal room of 3 by 3 metres, but you know, because there is what I can call inadequate monitoring or lack of monitoring, yes, there is particular number of people that should occupy a standard room—a maximum of two people, but in some cases four, five or more people occupy a single room in some of our major cities because of non-enforcement of housing standards.* (Urban planner, principal town planning officer, government)

*Most of the problems we have in the core congested areas … when you see 12–13 children living in two rooms … we say, ah, nothing is happening, about 6–7 people sleeping in a room, some sleeping outside; they don’t know they are killing themselves, even in planning we talk of high density. The maximum number of people that should sleep in a room is stated in the housing standards, but when it is getting to three or more people, it is becoming a problem.* (Urban planner, chief lecturer/consultant, government institution)

#### Achieving the buy-in of policy-makers

Another major barrier to the integration of FP and UD programmes was securing the buy-in of policy-makers in the relevant government agencies and ministries. It was reported that achieving multisectoral synergy is a policy issue that some of the respondents considered a herculean task, although not impossible. There may be the challenge of getting to connect the right people, and even in some instances, policy-makers demand bribes to get their attention and support. As an FP expert expressed:

*Well, it’s all about a policy issue. Actually, you need to work with policy-makers in both sectors to achieve the required synergy. … That will be the starting point…Yeah! Let me also tell you some of the challenges we faced in the process of translating our research findings into policy and laws. You see this xxx [referring to a top policy-maker in government], he is a friend of my son … So, everything I put forward to him he was calling every important person on my behalf. So, you need to know the right set of people. When the former xxx [referring to another top policy-maker] was there, he insisted we should go and bring ₦500,000*^2^* as a bribe before they can approve the policies and laws being canvassed for.* (FP expert, team lead, NGO)

There is also the challenge of the various professionals undermining each other’s contributions to policy-making, as the following excerpt indicates:

*We have to come together to a discussion table, maybe through dialogue and discussion that will bring us together so that we’ll understand and therefore create the desired synergy among different professions. If it’s an office where the family planning staff and the urban planners come together so that they’ll come together to understand themselves and the need to work together for the common benefit of the people, unless that is done, we’ll be looking at ourselves that we are not supposed to work together. The town planner understands that he needs the population; yeah, it is the area of family planning. Hmm, but, you see, the FP or healthcare expert may feel that he doesn’t need the town planner, but at the same time, the town planner may feel he doesn’t need the FP/healthcare information. Dialogue and roundtable discussion are important to achieve such synergy.* (Urban planner, principal lecturer, government institution)

#### Frequent transfer of government stakeholders

Another potential bottleneck to the integration of FP and UD programmes identified by respondents was the frequent transfer of government workers, especially the heads of units. This was considered a possible barrier because the frequent transfer of staff presents a challenge of having to deal with different people from time to time, some of whom might need training and retraining to understand the purpose and process of synergy and multisectoral actions. A respondent expressed the following view:

*You can come through the ministry, they know how to get people to do things, but the issue is that at times one might be trained, then maybe after 2 years or so …, you'll be taken to another department entirely so you have new people here now that would need to start afresh. This presents a challenge to multisectoral actions.* (FP expert, assistant chief nursing officer, government hospital)

#### Inadequate knowledge

Despite working with population figures for planning and development purposes, most urban planners do not know much about FP and its effect on population growth. Some urban planners reported that while they were aware of the existence of national and state FP blueprints, they did not have in-depth knowledge of its provisions. However, some others reported they did not know about these blueprints as they felt it was out of the scope of their profession, as they are urban planners, not health experts. Also, many of the FP experts had very limited knowledge about what urban planners do. The following extended excerpts bring the above issues to the fore:

*I’m not an expert in family planning …. I’m not a health planner. We have health planners, and they should be able to talk much about that.* (Urban planner, principal partner, private firm)

*I know there was just a time that there was a promotion of the use of condoms and contraceptives. And in my little interaction, we heard people who have said things about the pills, the injection and the side effects that they suffer…..Apart from that, I do not know much about family planning or anything about its policy.* (Urban planner, head, social entrepreneur, NGO)

*I don’t know urban planners, I don’t understand their work, and so I may not talk about synergy or collaboration with them until I know what they do, and how they fit into family planning.* (FP expert, executive director, CSO)

#### Perception about lack of nexus between FP and UD

Although most urban planners and FP experts expressed optimism and willingness to work together, one urban planner and one FP expert said they were unable to see a nexus or link between the two disciplines:

*That’s a very big one. I’ll say such synergy between FP and UD fields is unachievable…. I don’t think so, I don’t see it.* (Urban planner, consultant, government institution)

*In the FP programme, you consider population distribution, the high rate of population growth, and how to curb it. Definitely, you will get all this information without working with urban planners. There is no need for such synergy.* (FP expert, executive secretary, government)

### Multisectoral approach and collaborations between FP and UD experts

#### Intersectoral collaboration

Despite the recognized importance of population information to UD, there was little evidence of intersectoral synergy between FP and UD stakeholders, which was limited mainly to interagency collaborations among government ministries where meetings are held to consider issues of common interest such as population figures and estimates. The statements of experts below buttress this notion:

*No! I have never worked with urban planners. I think the set of people I have really worked with are the people in National Population Commission. We have worked together with those people several times, but have never worked with those in UD.* (FP expert, FP coordinator, government organization)

*Yeah, even though I know we have not been working with people in UD directly in our office, but there is a platform where we sometimes meet, most especially when we are talking about population.* (FP expert, RH/FP coordinator, government organization)

#### Suggestions on how to achieve multisectoral collaboration between FP and UD sectors

Despite reporting limited instances of previously working together, the stakeholders were open to collaboration but emphasized the need for a mutual understanding of the scope and operations of each profession. These issues are presented in the extended quotes below:

*We are open to any form of collaboration that will bring improvement to women’s health or adolescents’ health and even men’s health. So, yes, we are open to any collaboration with urban planners.* (FP expert, programme officer, NGO)

*Hmm, well, first the family planning experts need to understand the way the urban planners work in order to know where we can come in, and they too need to understand our work very well so that they will know how to work with us.* (FP expert, FP coordinator, government organization)

*We have to come together to a discussion table, maybe through dialogue and discussion that will bring us together so that we’ll understand and therefore create a common platform or an office where the family planning staff, as well as the urban planners, can work together. We know that we need synergy because of our common interests like population and the provisions of basic facilities.* (Urban planner, principal lecturer/consultant, government institution)

*Collaboration is possible when we begin to talk, when we begin to have programmes bringing us together, where you have urban planners coming to look at issues from their side and reproductive health/FP experts viewing the same issues from their side and then trying to marry the two together, and when we begin to do collaborative research. All those things will create a setting for a relevant policy to come out; that is when we can begin to see the synergy happening.* (Urban planner, principal consultant, government institution)

#### Ways to achieve synergy between FP and UD sectors

The respondents identified possible ways to achieve a synergistic working relationship between FP stakeholders and urban planners. The ways include involving nonprofessional stakeholders (such as the CSOs and the media), stakeholder engagement, roundtable discussion, interdisciplinary research, conferences and other interactive and knowledge-sharing fora, and sensitization talk with government officials and political leaders.

#### Stakeholder sensitization and engagement

Some urban planners and FP experts suggested that certain stakeholders in their professions (who work on policy formulation and those in charge of implementation and regulation) would need to come together to deliberate on multisectoral strategies that can be employed to ensure collaboration between FP and UD sectors. The excerpts below support this view:

*All professionals ... registered town planners—those in charge of policy formulation and regulation in the civil service, and those in academia and/or in practice should come together to have deliberations with the FP experts on possible multisectoral strategies. There is an important professional body; we call them APBN—Association of Professional Bodies in Nigeria—a conglomeration of all professionals that come together under one umbrella body to address issues that relate to all professional activities. They include town planners, surveyors, architects, doctors, lawyers and so on. That is a very good umbrella body that can serve as a forum for stakeholders’ engagement.* (Urban planner, acting director, government ministry)

*Essentially, stakeholders in one sector, say the FP field, need to reach out to professionals in another sector, like those in UD. Stakeholders in UD can also initiate the process of collaboration. That is the way to achieve synergy.* (FP expert, medical director, government hospital)

#### Involving nonprofessional stakeholders

Some of the respondents were of the view that the stakeholder engagement should be expanded to involve other actors such as the media, CSOs, faith-based organizations and others who are directly or indirectly involved in FP or UD, as two respondents put it:

*There are lots of superstitions around FP and procreation issues. Usually, government comes in, but we will not just leave it to the government … the media is critical too as a stakeholder in this. We will also need faith-based organizations because religion is playing a critical role in the issue of FP and population explosion. Civil society, traditional leaders and opinion leaders are critical too.* (Urban planner, head, social entrepreneur, NGO)

*You know, for the synergy to succeed, we must bring in everyone… All hands must be on deck. …, not just the health workers, but the leaders of thought like traditional leaders, Christian religious leaders and Islamic religious leaders must come in.* (FP expert, chief consultant gynaecologist, government hospital)

#### Conference and roundtable discussion

Other suggested strategies to achieve a viable collaboration include organizing a joint stakeholder conference for people from both professions to educate one another on FP and UD, and to discuss how they could achieve synergy between the two sectors. Also, there was the suggestion of interdisciplinary research which would lead to policy development. In line with this, some respondents were of the following view:

*When we work together, we will build a synergy that is likely to solve the problem than using a single sector approach to address issues because societal problems are multidimensional and if you don’t carry other relevant disciplines along you may be solving one problem while creating another one.* (Urban planner, university lecturer/NGO)

*We need to organize talks and see what urban planners do and also see what the family planning experts do and then create a common ground like implementing projects on family planning for the good of urban residents.* (FP expert, programme officer, NGO)

#### Sensitization talks with government officials and political leaders

A multisectoral synergy between FP and UD fields was considered a policy issue by some respondents. To these respondents, there is a need to create relevant policies to institutionalize the relationship. To achieve this, they suggested sensitization talks with policy-makers, as the following excerpt indicates:

*Hmm, sensitization!! The scheme is very important. We have to sensitize political actors and government officials. You know, they hardly have time to sit, but let them just sit down and we give them like 10-minute talk to sensitize them on the benefit of family planning for healthy urbanization.* (FP expert, regional coordinator, NGO)

## Discussion

The urban population continues to grow in Nigeria, with an annual urban growth rate of 6% [7]. To address the problem of unsustainable urbanization, a recent study by Harpham et al. [14] recognized the importance of multisectoral action and the need to bridge the gap between FP and UD programmes, while acknowledging that there are challenges to achieving this synergy. The present study explored barriers to achieving integrated policy formulation and implementation of FP and UD programmes in Nigeria. It further investigated how multisectoral actions and collaborations can be achieved between experts in the two fields.

Despite the recognized importance of the population factor to UD, as well as the value of multisectoral actions that guarantee efficient use of resources for good policy and programmatic outcomes as advanced by Harpham et al. [14] in their study, we found that there was little evidence of intersectoral collaboration between the stakeholders working in the FP and UD sectors. Our study found that there are some barriers to achieving synergy between the two sectors. We established that there were no identifiable relevant policies or programmes that explicitly link FP and UD. Scholars have argued that relevant policy and regulatory frameworks are critical to providing directions and guidance for the effective implementation of any programmes or interventions [18–20]. Although we found that stakeholders in the FP and UD sectors recognized the need for interagency collaborations to bridge the identified gaps, they also acknowledged the challenges of getting experts in the two fields to work together. A recent study indicates that differing political, historical and policy landscapes constitute cross-sectoral barriers that impede the synergistic working relationship between stakeholders in the fields of FP and UD [21].

The study established that there is a weak monitoring mechanism and poor implementation of existing strategies and programmes, particularly in the UD sector. We found that Nigeria’s UD sector is characterized by poor enforcement of UD and housing laws and regulations. Our results show that a major barrier to the effective implementation of UD is the failure on the part of the government’s statutory agencies to enforce relevant UD laws. We consider that for any meaningful and sustainable multisectoral collaboration to be achieved between FP and UD sectors, each field needs to put strategies in place for effective implementation of their various programmes. On one hand, existing policies, laws and regulations in each field must be effectively implemented and enforced. This is a necessary condition for successful synergy to be achieved. On the other hand, the problem of silos in research and policy/programmatic implementation in Nigeria and other African countries, which has created a huge interagency gap [22, 23], needs to be addressed to ensure the effective incorporation of FP programmes into the UD initiatives. Considering Nigeria’s rate of urbanization and its related issues such as urban poverty, poor urban waste management, unemployment, environmental degradation, inadequate infrastructure, and poor housing and overcrowding, there is a need to pursue multisectoral actions that will address the problem of unsustainable urbanization in the county. As scholars have noted [24, 25], there is a need to place the role of FP in curtailing the growth of urban centres on policy, programmatic and research agenda, not only in Nigeria but also in other high-fertility societies in low- and middle-income countries.

Further, the study found that securing the buy-in of relevant government agencies towards establishing the enabling regulatory framework and policy is a major barrier that needs to be addressed before successful multisectoral action can be achieved between the FP and UD sectors. Relevant stakeholders interviewed in both sectors contend that before any multisectoral collaboration or synergy can be achieved, pertinent policy and regulatory framework guiding such collaboration must be put in place. Many respondents noted that it may be difficult to achieve synergy between FP and UD sectors, particularly in northern Nigeria because of the negative perceptions about FP in that part of the country. However, one way of addressing this problem is to use an attractive entry point being advocated for in northern Nigeria such as labelling FP services as a child-spacing programme with the health benefits of reducing maternal and newborn mortality [25, 26].

Our study also established other barriers to the achievement of intersectoral synergy between FP and UD fields. These include stakeholder perception of the lack of nexus between the two sectors, and inadequate knowledge about policies and programmes that stakeholders consider to be out of their scope of profession or operation. For instance, FP experts know very little about what urban planners do, and vice versa, and as such may possibly not pursue any multisectoral actions between the two fields.

Notwithstanding the evidence of little synergy between the FP and UD sectors, we found that stakeholders in both fields were open to collaborations. They suggested some ways through which a synergistic working relationship could be established between FP stakeholders and urban planners. They emphasized the need for a mutual understanding of the operations of different professions. They identified the need for stakeholder engagement, interactions, knowledge-sharing, multidisciplinary research, workshops, conferences and roundtable discussions, as well as sensitization and publicity with relevant government agencies and other stakeholders. Respondents further suggested the need to pursue synergistic working relationships involving multiple stakeholders, not just FP experts and urban planners, but also other actors such as NGOs, civil societies, faith-based organizations, religious leaders and traditional leaders, as well as the media.

## Conclusion

The perspectives on how to achieve synergy between the FP and UD sectors as established in this study are not abstract notions; they derive from the experiences of relevant stakeholders working in applicable fields in both the private and public sectors. The selected professionals provided information on barriers and ways to achieve integrated policy formulation and implementation of FP and UD programmes in Nigeria. We found that relevant government agencies mostly work in silos. Other barriers to the synergy between the two sectors are lack of appropriate policy frameworks, ineffective implementation of guidelines in different sectors, and lack of knowledge about the FP–UD nexus among professionals. The study established ways to achieve synergy between FP and UD sectors, including stakeholder engagement, sensitization and publicity, roundtable discussion, interdisciplinary research, knowledge-sharing and intersectoral collaborations. The study also established that the frequent transfer of government employees is a barrier to achieving sustainable synergy between FP and UD sectors. We recommend that institutionalizing the implementation, monitoring and evaluation of FP in UD initiatives is fundamental to a sustained cross-sectoral synergy. Also, it is recommended that an UD programme curriculum review will be an appropriate effort towards mainstreaming FP into urban health programmes in Nigeria. The study concludes that addressing barriers to the intersectoral linkage between FP and UD is fundamental to achieving sustainable urbanization in Nigeria. It suggests the need for new sets of policies that prioritize FP in UD initiatives.

### Limitations

The study has some limitations. First, recall bias may be a concern due to the reliance on self-reporting. Second, the results may have some elements of social desirability bias for the same reason of self-reporting. However, measures were put in place during the fieldwork to minimize bias by ensuring the anonymity and confidentiality of solicited responses. Notwithstanding the study limitations, the study has addressed an important gap on how to ensure a multisectoral approach and collaborations between FP stakeholders and urban planners towards addressing the problem of urbanization and its concomitants in Nigeria.

## Data Availability

All data and materials to support the findings of this study are available from the corresponding author/principal investigator and will be made available upon reasonable request.

## Abbreviations

CSO: Civil society organization
FP: Family planning
GIS: Geographic information system
mCPR: Modern contraceptive prevalence rate
NGO: Nongovernmental organization
NURHI: Nigerian Urban Reproductive Health Initiative
RH: Reproductive health
SSA: Sub-Saharan Africa
SDG: Sustainable Development Goal
UD: Urban development
UNFPA: United Nations Population Fund

## Footnotes

1 Eight billion Nigerian naira was equivalent to US$ 20 million at the time of data collection.

2 ₦500,000 was the equivalent of US$ 1250.
